# Supplementary material for: A Survey on Knowledge, Attitude, and Practices of Large-Animal Farmers towards Antimicrobial Use, Resistance, and Residues in Mymensingh Division of Bangladesh
Source: Antibiotics (Basel). 2022 Mar 24;11(4):442. doi: 10.3390/antibiotics11040442 (PMC9030753; doi:10.3390/antibiotics11040442)
Supplement: Supplementary file 1 [file antibiotics-11-00442-s001.zip › antibiotics-1614470-supplementary.pdf]

S1: Questionnaire survey form regarding antibiotic usage pattern, its residual knowledge and antibiotic resistance (for Farmer)

A. General information (সাধারণ জ্ঞাতব্য)

1. Name of the farm (খামারের নাম): ....

2. Name of the owner/caretaker (মালিক/তত্ত্বাবধায়কের নাম): ....

....

3. Age of owner/caretaker (মালিক/তত্ত্বাবধায়কের বয়স): ....

4. Sex of owner/caretaker (মালিক/তত্ত্বাবধায়কের লিঙ্গ): ....

5. Address of the farm (খামারের ঠিকানা): ....

Phone (ফোন): .... E-mail (ই-মেইল): ....

6. Type of farm (খামারের ধরন): a) Cattle (গরু) [1] b) Goat (ছাগল) [2] c) Sheep (ভেড়া) [3] d) Buffalo (মহিষ) [4]

7. Total population (মোট সংখ্যা): ....

8. How many sheds in the farm (খামারে সেডের সংখ্যা): ....

9. Educational status of farm owner/caretaker (খামার মালিক/তত্ত্বাবধায়কের শিক্ষাগত যোগ্যতা):

a) Illiterate (অশিক্ষিত) [1] b) Primary (PSC) (প্রাথমিক/পিএসসি) [2] c) Junior Secondary (JSC) (জুনিয়র মাধ্যমিক/জেএসসি) [3]

d) Secondary (SSC) (মাধ্যমিক/এসএসসি) [4] e) HSC/Diploma (উচ্চ মাধ্যমিক/ডিপ্লোমা) [5] f) Graduate (স্নাতক) [6]

g) Masters (স্নাতকোত্তর) [7]

10. Receive any training program regarding antibiotic use and resistance from any institutions? (এন্টিবায়োটিকের ব্যবহার ও রেজিস্ট্যান্স সমন্ধে কোন প্রতিষ্ঠানের নিকট হতে প্রশিক্ষণে অংশগ্রহন করেছেন কি না)?

a) Yes (হ্যাঁ) 1 b) No (না) 0

B. Information about management practices in Livestock farms (খামারের ব্যবস্থাপনার চর্চা সম্পর্কে তথ্য)

1. Do you have any fence remains in the farm (আপনার খামারে বেড়া/প্রাচীর আছে)?

   a) Have (আছে) 1 b) Don't have (নাই) 0

2. Do you allow visitors in the shed (সেডে দর্শনার্থী প্রবেশের অনুমতি প্রদান করেন কি)?

   a) Yes (হ্যাঁ) 1 b) No (না) 0

3. Have any entrance of wild animal in the farm (খামারে বন্য প্রাণি প্রবেশ করে)?

   a) Yes (হ্যাঁ) 1 b) No (না) 0

4. Water supply (পানি সরবরাহ)

   a) Direct (সরাসরি) 1 b) Reserved (মজুদ রেখে) 0

5. Cleaning of waterer (পানির পাত্র পরিস্কার করা)

   a) Once daily (দিনে এক বার) 1 b) Once in a week (সপ্তাহে এক বার) 2

   c) Twice in a week (সপ্তাহে দুইবার) 3 d) Once in a month (মাসে এক বার) 4

   e) Twice in a month (মাসে দুইবার) 5

6. Supply of feed (খাদ্য সরবরাহ)

   a) Self processed (নিজ হাতে প্রস্তুতকৃত) 1 b) Purchased/Commercial (বাজার থেকে কেনা) 0

7. Cleaning of feeder (খাদ্যেও পাত্র পরিস্কার করা)

   a) Once daily (দিনে এক বার) 1 b) Once in a week (সপ্তাহে এক বার) 2

   c) Twice in a week (সপ্তাহে দুইবার) 3 d) Once in a month (মাসে এক বার) 4

   e) Twice in a month (মাসে দুইবার) 5

8. Cleaning of feces/floor/litter (মল-মুত্র/ফ্লোর/লিটার পরিস্কার করা)

   a) Once daily (দিনে এক বার) 1 b) Twice daily (দিনে দুইবার) 2

   c) Every alternate day (এক দিন পর পর) 3 d) Twice in a month (মাসে দুইবার) 4

   e) Once in a month (মাসে এক বার) 5 f) Once after sell 6

9. Do you have any footbath in your farm (খামারে ফুটবাথ আছে কি)?

   a) Yes (হ্যাঁ) 1 b) No (না) 0

10. How do you manage manure? (খামারে গোবরের ব্যবস্থাপনা কিভাবে করেন)

    a) Use as fertilizer (সার হিসেবে ব্যবহার) 1

b) Use for fuel (জ্বালানী হিসেবে ব্যবহার) 2

c) Sell for cash as fuel (জ্বালানী হিসেবে বিক্রী) 3

d) Leave on farm (খামারের মধ্যে ফেলে রাখা হয়) 4

e) Leave on open air (খোলা জায়গায় ফেলে রাখা হয়) 5

f) Discard into environment (পরিবেশে যত্রতত্র ফেলে রাখা হয়) 6

11. Vaccinate animals in schedule time (যথাসময়ে প্রাণিকে টিকা প্রদান করা হয়)?

a) Yes (হ্যাঁ) 1 b) No (না) 0

Farmers knowledge about antimicrobial use (AMU), Antimicrobial resistance (AMR) and antimicrobial residue (এন্টিমাইক্রোবিয়ালের ব্যবহার, এন্টিমাইক্রোবিয়াল প্রতিরোধী এবং এন্টিমাইক্রোবিয়ালের অবশিষ্টাংশ সমন্ধে খামারীর জ্ঞান):

K-1. Have you ever heard about antibiotic (আপনি কি এন্টিবায়োটিক সম্পকে শুনেছেন)?

a) Yes (হ্যাঁ) 1 b) No(না) 0

k-2. What antibiotic do (এন্টিবায়োটিক কি করে)?

a) Act against bacteria (ব্যাকটেরিয়ার বিরুদ্ধে কাজ করে) 1

b) Act against virus (ভাইরাসের বিরুদ্ধে কাজ করে) 2

c) Act against fungus/Others (ছত্রাক/অন্যান্য বিরুদ্ধে কাজ করে) 3

d) Act against all of the above 4

e) Don’t know (আমি জানি না) 5

K-3. Have you ever heard about antimicrobial resistance (আপনি কি জীবানু প্রতিরোধী সম্পর্কে শুনেছেন)?

a) Yes(হ্যাঁ) 1 b) No(না) 0

k-4. What do you know about antimicrobial resistance (আপনি জীবানু প্রতিরোধী সম্পর্কে কি জানেন)?

a) It causes treatment failure (এর কারণে চিকিৎসা ব্যর্থ হয়) 1

b) It causes poor response to treatment (এর কারণে চিকিৎসায় নগন্য সাড়া পাওয়া যায়) 2

c) Both (a+b) 3

d) Do not know 4

e) Others (অন্যান্য) 5

K-5. Do you know incomplete antibiotic course may lead to antibiotic resistance (আপনি জানেন কি, অসমাপ্ত এন্টিবায়োটিক কোর্স হতে এন্টিবায়োটিক প্রতিরোধী জীবানু তৈরী হতে পারে)?

1 0

a) Yes(হ্যাঁ) b) No(না)

K-6. Do you know overdose/low dose course may lead to antibiotic resistance (আপনি জানেন কি, উচ্চ মাত্রা/নিম্ন মাত্রায় এন্টিবায়োটিক ব্যবহার করলে এন্টিবায়োটিক প্রতিরোধী জীবানু তৈরী হতে পারে)?

a) Yes(হ্যাঁ) 1 b) No(না) 0

K-7. Have you ever heard about antibiotic residue (আপনিকি এন্টিবায়োটিক অবশিষ্ট সম্পর্কে শুনেছেন)?

a) Yes (হ্যাঁ) 1 b) No (না) 0

K-8. What is antibiotic residues? (এন্টিবায়োটিক অবশিষ্ট কি?)

a) Accumulation of antibiotics in human body through ingestion of meat and milk during antibiotic treatment (এন্টিবায়োটিক দিয়ে চিকিৎসারত প্রাণির দুধ/মাংস খাওয়ার পর মানুষের দেহে এন্টিবায়োটিক জমা হওয়া) 1

b) Accumulation of antibiotics in Animal body (প্রাণির দেহে এন্টিবায়োটিক জমা হওয়া) 2

c) Both (উভয়ই) 3

d) Don't know (আমি জানিনা) 4

K-9. Do you have any knowledge about biosecurity (আপনার কি জৈব নিরাপত্তা সম্পর্কে কোন জ্ঞান আছে)?

a) Have (আছে) b) Don't have (নাই)

K-10. Have you heard about withdrawal period of antibiotics (এন্টিবায়োটিকের অপসারণকাল সমন্ধে শুনেছেন কি? a) Yes (হ্যাঁ) 1 b) No (না) 0

K-11. Do you know antimicrobials have some side effects (আপনি জানেন কি এন্টিবায়োটিকের কিছু পার্শ্বপ্রতিক্রিয়া আছে)?

a) Yes (হ্যাঁ) 1 b) No (না) 0

Farmers attitude about antimicrobial use (AMU), Antimicrobial resistance (AMR) and antimicrobial residue (এন্টিমাইক্রোবিয়ালের ব্যবহার, এন্টিমাইক্রোবিয়াল প্রতিরোধী এবং এন্টিমাইক্রোবিয়ালের অবশিষ্টাংশ সমন্ধে খামারীর মনোভাব):

A-1. Use of same antibiotics to prevent any specific disease regularly give better result (কোন রোগ প্রতিরোধের জন্য নিয়মিত নির্দিষ্ট অ্যান্টিবায়োটিক ব্যবহার করলে ভাল ফল পাওয়া যায়)?

a) Yes (হ্যাঁ) 1 b) No (না) 0

A-2. Antimicrobials can be used to treat any kind of disease in animal (প্রাণির যে কোন রোগের চিকিৎসায় এন্টিবায়োটিক ব্যবহার হতে পারে)

a) Yes (হ্যাঁ) 1 b) No (না) 0

A-3. Stop antimicrobial treatment once animals feel better (প্রাণি সুস্থ বোধ করা মাত্রই এন্টিবায়োটিক চিকিৎসা বন্ধ করে দেন কি?)

a) Yes (হ্যাঁ) 1 b) No (না) 0

A-4. Better to sell animal products or slaughter animals during antimicrobial treatment or without maintaining withdrawal period in order to reduce cost of treatment (চিকিৎসা খরচ কমানোর জন্য এন্টিমাইক্রোবিয়াল দ্বারা চিকিৎসারত অবস্থায় প্রাণি জবাই/প্রাণিজাত খাদ্য বিক্রী করা ভাল)

a) Agree 1 b) Strongly agree 2 c) Disagree 3

A-5. To get better response alter the doses without consulting the prescribers (আপনি কি প্রেসক্রিপশনকারীকে না জানিয়ে ঔষধের মাত্রা পরিবতন করেন ভাল সাড়া পাওয়ার জন্য?)

a) Yes (হ্যাঁ) 1 b) No (না) 0

A-6. Use of antimicrobials may be reduced by maintaining proper biosecurity, vaccination and good management (সঠিক বায়োসিকিউরিটি মেনে চলে, নিয়মিত টিকা প্রদানের মাধ্যমে এবং ভাল ব্যবস্থাপনার দ্বারা এন্টিমাইক্রোবিয়ালের ব্যবহার কমানো সম্ভব)

a) Yes (হ্যাঁ) 1 b) No (না) 0

A-7. Antibiotics be used only when needed

a) Agree 1 b) Strongly agree 2 c) Disagree 3

A-8. Antibiotics should be prescribed only by veterinarians?

a) Agree 1 b) Strongly agree 2 c) Disagree 3

A-9. Use antibiotics as growth promoters is necessary in livestock production

a) Agree [1] b) Strongly agree [2] c) Disagree [3]

Farmers Practice about antimicrobial use (AMU), Antimicrobial resistance (AMR) and antimicrobial residue (এন্টিমাইক্রোবিয়ালের ব্যবহার, এন্টিমাইক্রোবিয়াল প্রতিরোধী এবং এন্টিমাইক্রোবিয়ালের অবশিষ্টাংশ সমন্ধে খামারীর চর্‌চা/অনুশীলন):

P-1. Use of antibiotics recommended by (কার পরামর্শে অ্যান্টিবায়োটিক ব্যবহার করেন)?

Put 1 if answer is Yes, otherwise put 0

a) Veterinarian (ভেটেরিনারিয়ান) [1]
b) Other farmers (অন্য খামারি) [1]
c) Shopkeeper (দোকানদার) [1]
d) Representative of pharmaceutical company (ঔষধ কোম্পানির প্রতিনিধি) [1]
e) Veterinary Paraprofessional (ভেটেরিনারিয়ানের সাহায্যকারী) [1]
f) Village Doctor/ Quack (গ্রাম্য/হাতুড়ে প্রাণি চিকিৎসক) [1]
g) Self (নিজ) [1]

P-2. Do you keep record of using antimicrobials (আপনি এন্টিমাইক্রোবিয়াল ব্যবহারের রেকর্ড রাখেন)?

Put the code of respective answer (any one)

a) Always (সর্বদা) [1]
b) Most frequently (প্রায়সময়ই) [2]
c) Sometimes (কোন কোনসময়) [3]
d) Rarely (কদাচিত) [4]
e) Never (কখনো না) [5]
f) Do not know (জানি না) [6]

P-3. Antibiotic courses completed last time (সর্বশেষ ক্ষেত্রে এন্টিবায়োটিক কোর্স সম্পন্ন করেছেন কিনা)?

a) Yes (হ্যাঁ) [1] b) No (না) [0]

P-4. Number of antibiotics used at a time in your farm (একই সময় অ্যান্টিবায়োটিক ব্যবহারের সংখ্যা)

a) Single (একক ভাবে) [1] b) Combined/Multiple (একাধিক) [2]
c) both (a+b) (উভয়ই) [3] d) Do not know (জানিনা) [4]

P-5. Withdrawal period follows (উইথড্রয়াল সময়ের অনুসরণ)?

a) Yes (হ্যাঁ) [1] b) No (না) [0]

P-6. Do you add antibiotics to the animal feed (আপনি প্রাণিখাদ্যে এন্টিবায়োটিক মেশান কি না)?

a) Yes (হ্যাঁ) [1] b) No (না) [0]

P-7. Storage of drug (ঔষধ সংরক্ষণ)

a) Store room (স্টোর রুম) [1] b) Refrigerator (ফ্রিজ) [2]

c) Shed (সেড) [3] d) Bedroom/Others (শয়নকক্ষ/অন্যান্য) [4]

P-8. Do you follow the exact prescription of Veterinarian during purchasing antibiotic (সঠিক এন্টিবায়োটিক কেনার ক্ষেত্রে ভেটেরিনারিয়ান এর প্রেসক্রিপশন হুবহু অনুসরন করেন)?

a) Always (সব সময়) [1]

b) Sometimes influenced by medicine seller/others (দোকানদার বা অন্যান্য কর্তৃক পরিবর্তন) [2]

P-9. What do you do with leftover antibiotics (ব্যবহারের পর অতিরিক্ত এন্টিবায়োটিক আপনি কি করেন?

a) Keep for further use (পরবর্তীতে ব্যবহারের জন্য রেখে দেন)? [1]

b) Through in the garbage (ময়লা রাখার স্থানে ফেলে দেন)? [2]

c) Give it to other farmers for use (অন্য খামারীকে ব্যবহারের জন্য দিয়ে দেন)? [3]

d) Burry in the ground/Burn (মাটিতে পুতে ফেলেন/পুড়িয়ে ফেলেন)? [4]

P-10. Do you read the prospectus before using antimicrobials? (আপনি কি এন্টিবায়োটিক ব্যবহারের পূর্বে নির্দেশনাবলী পড়েন?)

a) Yes (হ্যাঁ) [1] b) No (না) [0]

Additional question to farmers (খামারীদের নিকট অতিরিক্ত প্রশ্নাবলী)

1. Please mention the name of common antibiotics you frequently use in livestock

(যেসকল অ্যান্টিবায়োটিক প্রায়ই আপনার প্রাণীতে ব্যবহার করেন, সেগুলোর নাম লিখুন)

a) Penicillin (পেনিছিলিন) [1]

b) Tetracycline (টেট্রাসাইক্লিন) [1]

c) Doxycycline (ডক্সিসাইক্লিন) [1]

d) Oxytetrecycline (অক্সিটেট্রাসাইক্লিন) [1]

e) Streptomycin (স্ট্রেপ্টোমাইসিন) [1]

Put 1 if answer is Yes, otherwise put 0

f) Gentamycin (জেন্টামাইসিন) 1

g) Cephalexine (সেফালেক্সিন) 1

h) Ceftriaxone (সেফট্রিয়াক্সোন) 1

i) Ciprofloxacin (সিপ্রোফ্লোক্সাসিন) 1

j) Cloxacillin (ক্লোক্সাসিলিন) 1

k) Cefixime (সেফিক্সিম) 1

l) Sulphar drug (Combined) (সালফার ড্রাগ-কম্বাইন্ড) 1

m) Sulfadimidine (সালফাডিমিডিন) 1

n) Sulfadiazine (সালফাডায়াজিন) 1

o) Sulphamethoxazole (সালফামিথোক্সাজল) 1

p) Ampicillin (এম্পিসিলিন) 1

q) Amoxycillin (এমোক্সিসিলিন) 1

r) Ceftiofur (সেফটিওফার) 1

s) Lincomycin (লিনকোমাইসিন) 1

t) Azithromycin (এজিথ্রোমাইসিন) 1

u) Amikacin (এমিকাসিন) 1

v) Others (অন্যান্য) 1

2. How antibiotic treatments affects the economy of your farm (এন্টিবায়োটিকের ব্যবহার কিভাবে খামারের অরথোনীতিকে প্রভাবিত করে)

a) Heavily (প্রবলভাবে) b) Lightly (হালকাভাবে) c) doesn’t affect at all (কোন প্রভাব পড়ে না)

3. Causes of not following withdrawal period (উইথড্রয়াল পিরিয়ড ফলো না করার কারণসমূহ)

a) To minimize economic loss (আর্থিক ক্ষতিপুরন করতে) 1

b) Prescriber not even suggest to follow (প্রেশক্রিপশনকারীরা কেউ বলে না) 2

(c) Don’t know about withdrawal pediod (উইথড্রয়াল পিরিয়ড সমন্ধে জানি না). 3
